# Inhibition of myocyte-specific enhancer factor 2A improved diabetic cardiac fibrosis partially by regulating endothelial-to-mesenchymal transition

**DOI:** 10.18632/oncotarget.8842

**Published:** 2016-04-20

**Authors:** Xue-ying Chen, Rui-juan Lv, Wei Zhang, Yu-gang Yan, Peng Li, Wen-qian Dong, Xue Liu, Er-shun Liang, Hong-liang Tian, Qing-hua Lu, Ming-xiang Zhang

**Affiliations:** ^1^ The Key Laboratory of Cardiovascular Remodeling and Function Research, Chinese Ministry of Education and Chinese Ministry of Public Health, Qilu Hospital of Shandong University, Jinan, Shandong, China; ^2^ Department of Emergency, Qilu Hospital of Shandong University, Jinan, Shandong, China; ^3^ College of Pharmacy, Xinxiang Medical University, Xinxiang, China; ^4^ Department of Medicinal Chemistry, School of Pharmacy, Shandong University, Jinan, Shandong, China; ^5^ Department of Cardiology, The Second Hospital of Shandong University, Jinan, Shandong, China

**Keywords:** myocyte-specific enhancer factor 2A, fibrosis, diabetes mellitus, endothelial mesenchymal transition

## Abstract

Cardiac fibrosis is an important pathological process of diabetic cardiomyopathy, the underlying mechanism remains elusive. This study sought to identify whether inhibition of Myocyte enhancer factor 2A (MEF2A) alleviates cardiac fibrosis by partially regulating Endothelial-to-mesenchymal transition (EndMT). We induced type 1 diabetes mellitus using the toxin streptozotocin (STZ) in mice and injected with lentivirus-mediated short-hairpin RNA (shRNA) in myocardium to inhibit MEF2A expression. Protein expression, histological and functional parameters were examined twenty-one weeks post-STZ injection. We found that Diabetes mellitus increased cardiac MEF2A expression, aggravated cardiac dysfunction and myocardial fibrosis through the accumulation of fibroblasts via EndMT. All of these features were abolished by MEF2A inhibition. MEF2A gene silencing by shRNA in cultured human umbilical vein endothelial cells (HUVECs) ameliorated high glucose–induced phenotypic transition and acquisition of mesenchymal markers through interaction with p38MAPK and Smad2. We conclude that inhibition of endothelial cell-derived MEF2A might be beneficial in the prevention of diabetes mellitus-induced cardiac fibrosis by partially inhibiting EndMT through interaction with p38MAPK and Smad2.

## INTRODUCTION

Diabetes mellitus (DM) can affect cardiac structure and function and lead to heart failure in the absence of atherosclerosis and hypertension, which is called diabetic cardiomyopathy (DCM) [1]. Myocardial fibrosis is often present in end-stage heart failure caused by DCM. Although fibrosis, which is attributed to an excess deposition of extracellular matrix (ECM) components, is one of the most common pathological changes found in various organs, including the heart, the detailed mechanism responsible for this change remains unclear. Given the increased risk of heart failure in diabetic patients, a better understanding of the underlying mechanisms and additional therapeutic strategies would be of considerable value.

Studies have shown that endothelial-to-mesenchymal transition (EndMT) plays an important role in myocardial fibrosis [2]. EndMT is considered to be a driving process resulting in the trans-differentiation of endothelial cells into mesenchymal cell types, characterized by a loss of cell-cell adhesion and a change in cell polarity and accompanied by a reduction in endothelial marker expression, such as vascular endothelial cadherin (VE-cadherin) and CD31, and an increase in mesenchymal marker expression, including fibroblast-specific protein-1 (FSP-1), α-smooth muscle actin (α-SMA), and vimentin [3] and so on. EndMT is stimulated by TGF-β2 through Smad, MEK (MAPK [mitogen-activated protein kinase]/ERK [extracellular signal-regulated kinase]), PI3K (phosphoinositide3-kinase), and p38 MAPK signaling pathways. Inhibitors of these pathways prevent TGF-β2-induced EndMT [4]. Increasing evidence has shown that high glucose levels can induce EndMT [5–7]. However, the factors regulating EndMT under pathologic conditions of high glucose are not clear and remain to be elucidated.

Myocyte enhancer factor 2A (MEF2A) belongs to a family of four closely related transcription factors (MEF2A, -B, -C, and -D) that are conserved from yeast to humans [8]. MEF2A functions during fetal development of the cardiovascular system and controls cell proliferation, differentiation, and death in both the developing fetus and the adult [3]. Recent reports [9, 10] have provided evidence of a role for hepatic stellate cell and myocardial cell MEF2A in fibrosis, although the precise functions of this transcription factor in endotheliocyte are still unclear. MEF2A is expressed in endothelial cells and is closely associate with angiogenesis [11]. The overall expression pattern of MEF2A is similar to vascular endothelial growth factor receptor 2(VEGFR2) and Von Willebrand factor [12].

In addition, some studies indicated that bone morphogenetic protein2 (BMP-2), Smad2 [13], MAPKs p38 and ERK5 [14] interacted with MEF2A. Meanwhile, these proteins are important signal molecules of signaling pathways, which regulate EndMT. Therefore, we hypothesized that administration of MEF2A might achieve cardioprotective effects against fibrosis in diabetic hearts partially by regulating EndMT. To support our hypothesis, we performed a series of experiments both *in vivo* and *in vitro*.

## RESULTS

### Diabetes increases myocardial MEF2A expression and MEF2A inhibition attenuates diabetes-induced cardiac dysfunction

**Figure 1:**
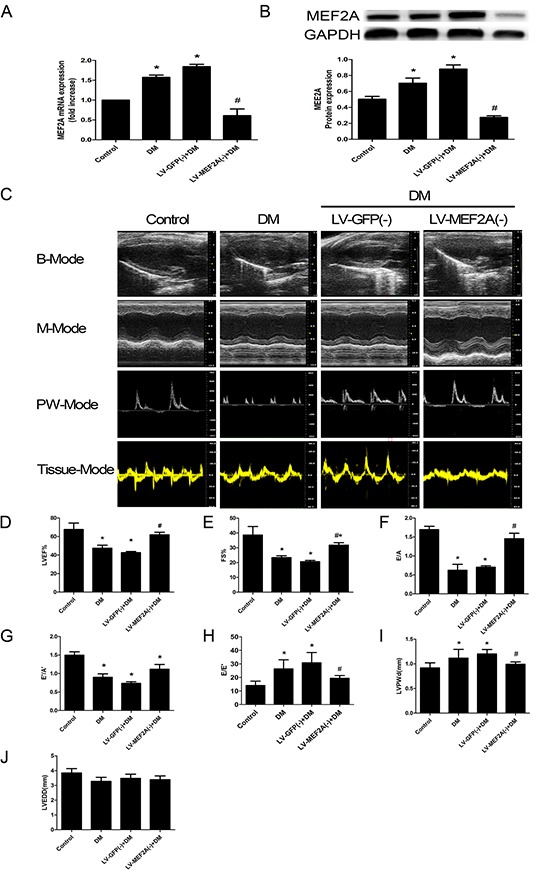
MEF2A expression and Echocardiography measurements of mouse hearts **A.** Quantitative RT-PCR analysis of MEF2A mRNA expression; **B.** Western blot analysis of MEF2A in hearts from all groups; **C.** Typical echocardiographic images of 2D echocardiograms, M-mode echocardiograms, pulsed-wave Doppler echocardiograms and tissue Doppler echocardiograms in all groups; **D**–**J.** Quantitative analysis of echocardiographic measurements parameters. **(D)** Left ventricular ejection fraction (LVEF). **(E)** Fractional shortening (FS). **(F)** Early to late mitral flow (E/A). **(G)** Early to late ratio of diastolic mitral annulus velocities (E'/A'). **(H)** Early mitral flow ratio to tissue Doppler-derived early mitral annulus velocity (E/E'). **(I)** Left ventricular posterior wall thickness at diastole (LVPWd). **(J)** Left ventricular end-diastolic dimension (LVEDD). Data are mean±SEM. *p<0.05 compared with control; # p<0.05 compared with vehicle treatment group. n= 8 to 10 mice for each group.

STZ induced rapid hyperglycemia in mice compared with the citrate treatment beginning 1 week after injection. Blood glucose, body weight, heart weight, and the ratio of heart weight to body weight were comparable among the 4 groups twenty-one weeks post-STZ injection. Body weight (g) and heart weight (mg) were lower in diabetic mice than in control mice (18.60±0.59 vs. 28.94±0.96, 102.50±2.94 vs. 124.54±4.40, respectively; *p*<0.05). However, the ratio of heart weight to body weight (mg/g) (5.52±0.28 vs. 4.30±0.61, *p*<0.05) was significantly higher in diabetic mice than in control mice (Table 1). Diabetic mice showed increased myocardial MEF2A mRNA levels (1.57±0.10-fold increase) and protein levels (1.40±0.02-fold increase) as compared with healthy controls (Figure 1A and 1B). Treatment with shRNA-MEF2A downregulated myocardial MEF2A protein levels (3.21±0.25-fold decrease, *p*<0.05) compared with vehicle treatment in diabetic mice (Figure 1B). Heart weight (90.70±7.10 vs. 101.10±12.12, *p*<0.05) and the ratio of heart weight to body weight (5.07±0.90 vs. 5.50±0.68, *p*<0.05) were lower with shRNA-MEF2A treatment than vehicle treatment (Table 1). ShRNA-MEF2A and vehicle-transfected diabetic mice did not significantly differ in body weight (17.89±0.83 vs. 18.33±0.60, p>0.05; Table 1). Compared with control mice, diabetic mice also showed cardiac structure change and cardiomyocyte width increased, and MEF2A silencing attenuated the change compared with vehicle treatment (Supplementary Figure S2). These results suggested that MEF2A inhibition could reverse cardiac remodeling in diabetic mice.

**Table 1:** Measurements of blood glucose, body weight, heart weight, and HW/BW

Group	BG (mM)	BW (g)	HW (mg)	HW/BW(mg/g)
Control	9.40±1.37	28.94±0.96	124.54±4.40	4.30±0.61
DM	36.40±1.39*	18.60±0.59*	102.50±2.94*	5.52±0.28*
LV-GFP(−)+DM	40.58±4.56*	18.33±0.60*	101.10±12.10*	5.50±0.68*
LV-MEF2A(−)+DM	34.95±1.78*	17.89±0.83*	90.70±7.10* †	5.07±0.90* †

BG indicates blood glucose, BW indicates body weight, HW indicates heart weight; all results are presented as mean±SEM.

* *p*< 0.05 vs. control group; †*p*< 0.05 vs. vehicle control. Control: normal mice; DM: diabetic cardiomyopathy group; LV-GFP (−): MEF2A-LV interference vehicle treatment; LV-MEF2A (−): MEF2A-LV interference treatment.

Echocardiogram was performed to assess the cardiac function at 20 weeks after STZ injection. At baseline, LVEF, FS, E/A, E'/A' and E/E' ratios, LVPWd and LVEDD did not differ among groups (data not shown). After twenty-one weeks post-STZ injection, echocardiography revealed poor values for cardiac performance, including LVEF, FS, E/A, E'/A' and E/E' ratios (Figure 1C). Cardiac function features were lower in diabetic than control mice: LVEF (47.47±5.65% vs. 67.62 ±6.9%, p<0.05) (Figure 1D), FS (23.34±2.64% vs. 38.60±5.74%, p<0.05) (Figure 1E), E/A ratios (0.62±0.31 vs. 1.70±0.23, p<0.05) (Figure 1F), E'/A' ratios (0.90±0.16 vs. 1.50±0.24, p<0.05) (Figure 1G) and E/E' ratios (26.30±6.72 vs. 14.04±3.25, p<0.05) (Figure 1H). As compared with vehicle treatment, MEF2A-LV interference treatment improved the attenuated LVEF (61.90±4.68% vs. 42.61±2.14%, p<0.05) (Figure 1D), FS (31.79±2.85% vs. 20.62±1.46%, p<0.05) (Figure 1E), E/A ratios (1.45±0.25vs 0.70±0.06, p<0.05) (Figure 1F), E'/A' ratios (1.12±0.23 vs. 0.73±0.08, p<0.05) (Figure 1G) and E/E' ratios (19.41±2.00 vs. 30.82±7.58, p<0.05) (Figure 1H) in diabetic mice. To test whether high-glucose induced cardiac hypertrophy, LVPWd, LVEDD were examined. LVPWd was significantly increased in diabetic mice (1.12±0.35 vs. 0.92±0.27, p<0.05), and MEF2A-LV interference treatment attenuated the wall thickness (0.99±0.08 vs. 1.20±0.16, p<0.05) (Figure 1I). LVEDD in DM group decreased, whereas there was no statistically significant difference (p>0.05) (Figure 1G).

### MEF2A inhibition limits diabetes-induced myocardial fibrosis

**Figure 2:**
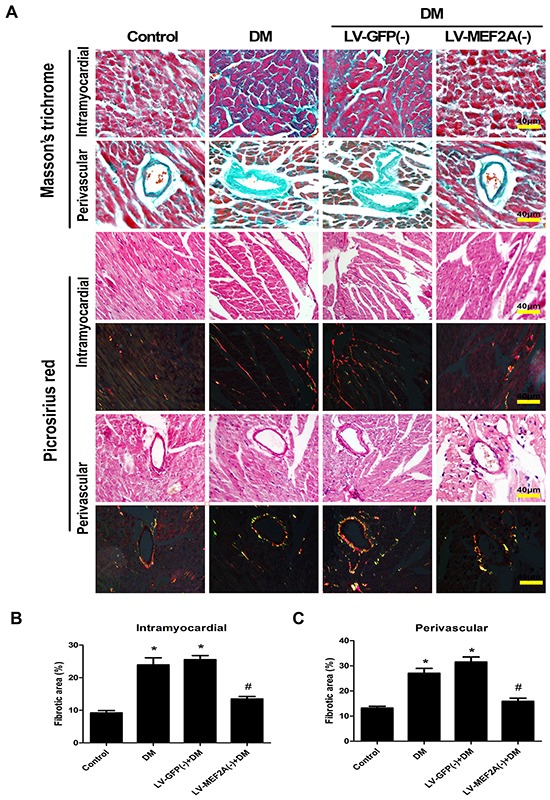
MEF2A inhibition limits diabetes-induced myocardial fibrosis **A.** Interstitial and perivascular fibrosis were analyzed by Masson's trichrome (blue staining) and Picrosirius Red staining (red and yellow staining) after 20 weeks of diabetes mellitus in all groups mouse. Scale bars=40 μm. **B.** Quantification of interstitial fibrosis after 20 weeks of diabetes mellitus. **C.** Quantification of perivascular fibrosis after 20 weeks of diabetes mellitus. Data are mean±SEM. *p<0.05 compared with control; # p<0.05 compared with vehicle treatment group. n= 8 mice for each group.

Masson's trichrome and Picrosirius red staining of heart sections revealed an increase in ECM in the perivascular and intramyocardial regions of the diabetic mouse myocardium (Figure 2A). Quantitative analysis of Masson' trichrome staining showed that diabetic mice had a 2.60- and 2.05-fold increase in collagen deposition in the intramyocardial and perivascular regions as compared with control mice (23.91±5.84% vs. 9.19±1.62%, *p*< 0.05; and 27.04±5.09%, vs. 13.19±1.62%, *p*<0.05). ShRNA-MEF2A treatment reduced collagen deposition as compared with vehicle treatment (13.46±2.07% vs. 25.55±3.27%, *p*<0.05; and 15.88±3.33%, vs. 31.51±4.89%, *p*< 0.05; Figure 2B and 2C).

**Figure 3:**
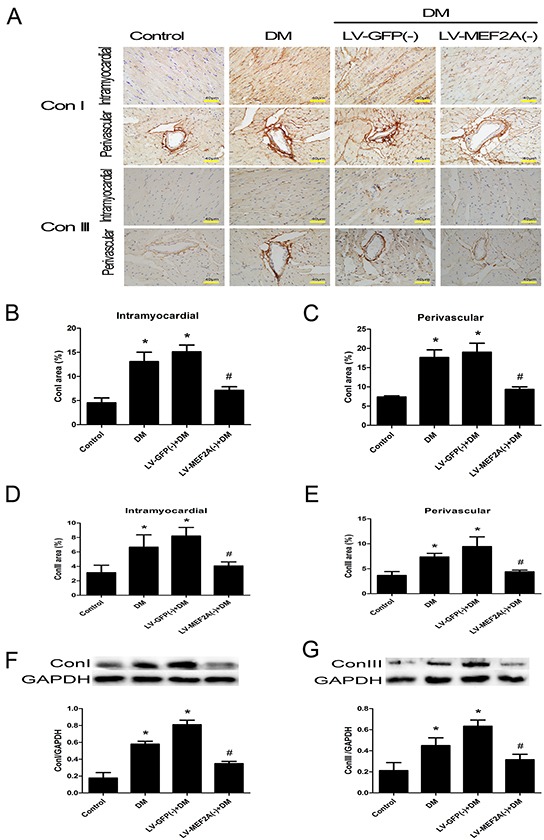
MEF2A inhibition limits diabetes-induced collagen deposition in a diabetic mouse model **A.** Immunostaining of collagen I and III in the interstitial and perivascular region, respectively (scale bars=40 μm); **B.** and **C.** Quantification analysis of collagen I in the interstitial and perivascular region; **D.** and **E.** Quantification analysis of collagen III in the interstitial and perivascular region, respectively; **F.** and **G.** Western blot analysis of collagen I and collagen III; Data are mean ± SEM. *p< 0.05 compared with control; #*p*< 0.05 compared with vehicle treatment group; n= 8 mice for each group.

Diabetes enhanced the expression of fibrotic markers collagen I and III as compared with the control group, whereas shRNA-MEF2A transfection in diabetic mice significantly reduced collagen levels as compared with vehicle treatment (Figure 3A–3E). The immunohistochemistry data were confirmed by western blot analysis results (*p*< 0.05; Figure 3F and 3G).

### MEF2A knockdown inhibits myocardial fibrosis partially by suppressing EndMT *in vitro* and *in vivo*

To determine whether MEF2A inhibits high glucose (HG)-induced EndMT, we performed *in vitro* and *in vivo* experiments.

**Figure 4:**
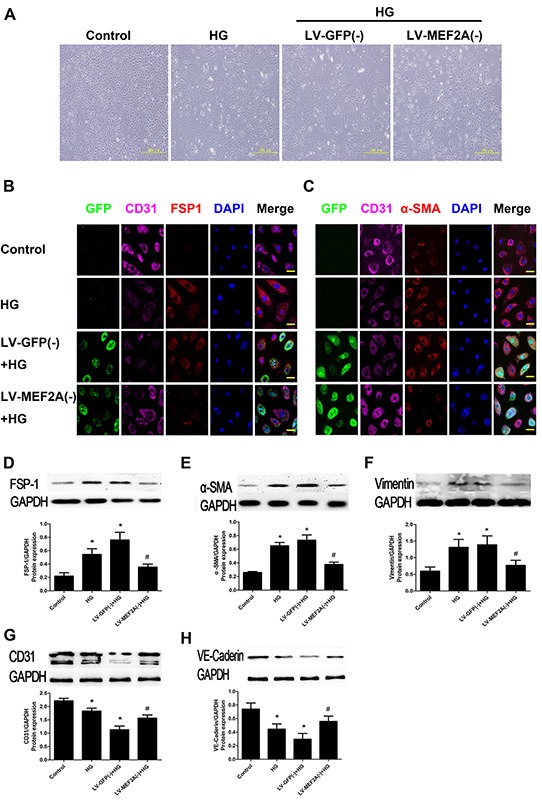
MEF2A inhibition limits HG-induced EndMT in HUVECs **A.** The morphological change in HUVECs after HG treatment with or without MEF2A knockdown, resulting in a fibroblast-like spindle-shaped form. Scale bars=200 μm. **B.** Double immunofluorescence staining with antibodies to CD31 (Purple) and S100A4/FSP-1(Red). **C.** Double immunofluorescence staining with antibodies to CD31 (Purple) and α-SMA (Red); GFP (Green) was label of lentivirus and determine which cells had been transfected. Nuclei were counterstained with DAPI (blue). Scale bars=40μm. Results are from 3 repeated experiments. **D–H.** MEF2A inhibition decreased the protein expression of S100A4/FSP-1, α-SMA, vimentin, and increased CD31, VE-cadherin protein expression. Control: 5 mmol/l glucose; HG: 33 mmol/l glucose; LV-GFP (−): MEF2A-LV interference vehicle treatment; LV-MEF2A (−): MEF2A-LV interference treatment; Data are mean ± SEM. *p<0.05 compared with control; #p<0.05 compared with vehicle treatment group. Results are from 3 repeated experiments.

*In vitro*, HUVECs were treated with 33 mmol/L d-glucose with or without ShRNA-MEF2A for 5 days. Fluorescence microscopy revealed that control HUVECs showed a typical rounded or cobblestone shapes, and MEF2A silencing inhibited the change from a cobblestone-like to spindle-shaped feature with HG induction (Figure 4A). Immunofluorescence and western blot analysis demonstrated that HG-treatment significantly increased the levels of mesenchymal markers FSP-1, α-SMA, and vimentin and caused a reduction in the levels of endothelial markers CD31 and VE-Cadherin as compared with controls. However, the features above were alleviated when MEF2A was knocked down. GFP was used to evaluate transfection efficiency, which reached values of up to 90% (Supplementary Figure S1B). Immunofluorescence-colocalized staining showed that MEF2A silencing reduced expression of S100A4/FSP1 and α-SMA and increased expression of CD31. In the MEF2A knockdown group, the cells transfected by GFP had weak α-SMA expression, while those not transfected by GFP had strong α-SMA expression (Figure 4B and 4C). The effect of MEF2A on HG-induced EndMT was also detected by western blot. Mesenchymal markers (FSP-1, α-SMA, and vimentin) increased in the HG-group compared with the control group, and knockdown of MEF2A caused them to decrease (Figure 4D–4F). However, the opposite results were seen for the endothelial markers (CD31 and VE-Cadherin; Figure 4G–4H).

**Figure 5:**
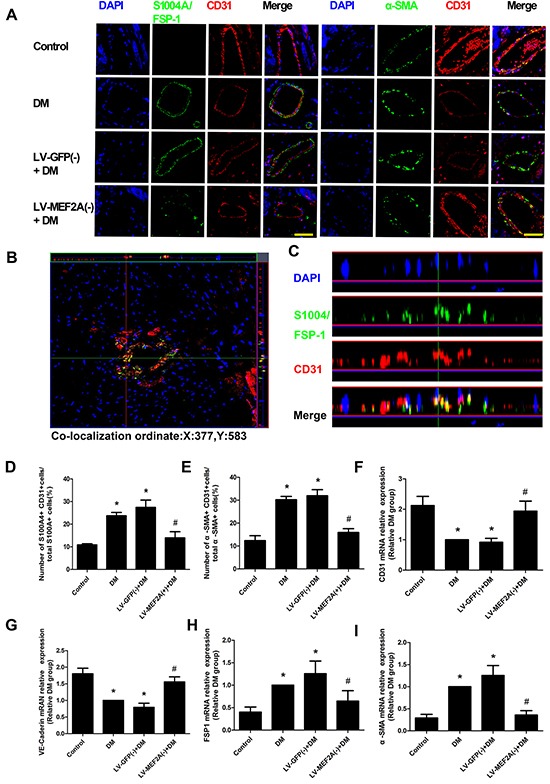
MEF2A inhibition limits cardiac EndMT in a diabetic mouse model **A.** Double immunofluorescence staining of antibodies to CD31 (Red) with antibodies to S100A4/FSP-1(Green) and α-SMA (green) in coronary arterioles of all groups mouse. Colocalization of CD31 with S100A4/FSP-1 and α-SMA expression in coronary arterioles is shown in yellow. DAPI (Blue) was used to stain nucleus. Scale bars=40 μm. **B.** and **C.** Representative z-stack image analysis shows specific overlay of double immunostaining, CD31+/S100A4+ cells in specific ordinate were analyzed in z stack with optimal interval range of 0.8μm. **D.** and **E.** The percentage of S100A4+ CD31+ cells and α-SMA+ CD31+ cells in diabetic hearts. **F–I.** RT-PCR analysis shows CD31, VE-Cadherin, FSP-1 and α-SMA in sorted cardiac endothelial cells by magnetic affinity cell sorting using a CD146 antibody. Data are mean ± SEM. *p<0.05 compared with control; #p<0.05 compared with vehicle treatment group. n=12 mice for each group.

*In vivo*, we performed double immunofluorescence staining with antibodies to CD31 and S100A4/FSP-1. We observed colocalization of CD31 and S100A4/FSP-1 expression in the endothelial layer of interstitial tissue and microcapillary vessels. Other mesenchymal cell markers, such as α-SMA, also showed colocalization with CD31+ cells (Figure 5A). The double-positive immunofluorescence staining was examined by z-stack image analysis, which confirmed the specific overlay of CD31+/ FSP-1+ cells (Figure 5B and 5C), which showed that CD31+/ FSP-1+ cells and CD31+/α-SMA+ cell numbers is significantly increased in the diabetic mice compared with the control mice (23.47±7.25% vs. 10.28±1.76% and 30.12±8.11% vs. 13.18±2.13%, respectively; *p*< 0.05). Moreover, the shRNA-MEF2A transfection in diabetic mice significantly reduced the levels as compared with vehicle treatment (14.39±3.8% vs. 28.61±7.54% and 16.54±2.18% vs. 33.09±8.93%, respectively; *p*< 0.05; Figure 5D and 5E). The immunohistochemistry data were confirmed with RT-PCR analysis results in cardiac endothelial cells by magnetic affinity cell sorting using a CD146 antibody (Figure 5F–5I).

### HG-induced EndMT is mediated by the translocation of MEF2A to the cytoplasm and interactions with p38MAPK and Smads in HUVECs

**Figure 6:**
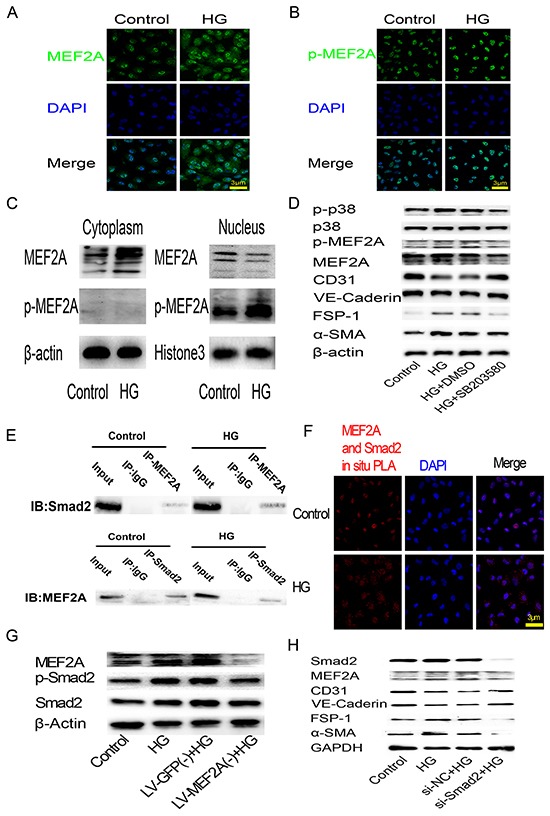
HG-induced EndMT is mediated by the translocation of MEF2A to the cytoplasm and interactions with p38 MAPK and smads in HUVECs **A.** and **B.** Immunofluorescence analysis of MEF2A and phosphorylated-MEF2A localization in HUVECs. MEF2A is stained green, nuclei are stained blue with DAPI (scale bar =3 μm); **C.** Western blot analysis of MEF2A and phosphorylated-MEF2A expression levels in cytoplasm and nuclear proteins of HUVECs; **D.** Western blot analysis of phosphorylated-p38, p38, phosphorylated-MEF2A, MEF2A, CD31, VE-Cadherin, FSP-1 and α-SMA in HUVECs after HG treatment with or without SB203580; **E.** CO-IP analysis of the protein-protein interaction between MEF2A and Smad2. **F.** PLA analysis of the protein-protein interaction between MEF2A and Smad2. The purple-stained dots represent MEF2A/Smad2-binding proteins. Nuclei are stained blue with DAPI (scale bar =3μm); **G.** Western blot analysis of MEF2A, phosphorylated-Smad2 and Smad2in HUVECs after HG treatment with or without MEF2A knockdown; **H.** Western blot analysis of Smad2, MEF2A, and EndMT markers in HUVECs after HG treatment with or without Smad2 knockdown by siRNA.

Immunofluorescence and western blot analysis revealed HG induced relocation of MEF2A in the cytoplasm of HUVECs, but the phosphorylation state of MEF2A located in the nucleus consistently and HG induced phosphorylation of MEF2A increasing (Figure 6A–6C). Conversely, Western blot analysis showed inhibition of phosphorylation of p38 by inhibitor SB203580 was sufficient to prevent the increase of MEF2A, p-MEF2A, FSP-1,α-SMA, and the decrease of CD31, VE-cadherin induced by HG (Figure 6D).

To study the interaction between MEF2A and Smad2, CO-IP and PLA was performed to image protein-protein interaction between MEF2A and Smad2. Co-Immunoprecipitation (CO-IP) results showed there was relationship between the MEF2A and Smad2 (Figure 6E). Interestingly, PLA showed that the dots representing MEF2A/Smad2-bingding proteins tended to localize in the nucleus in control group, and localized in the cytoplasm in HG group (Figure 6F). Western blot analysis showed that HG increase Smad2 phosphorylation in HUVECs as compared with control group, while MEF2A knockout made Smad2 phosphorylation declined (Figure 6G). Conversely, MEF2A expression decreased after smad2 knockdown by si-RNA. FSP-1 and α-SMA decreased, and CD31 and VE-Cadherin increased after smad2 knockdown before HG treatment (Figure 6H).

## DISCUSSION

DM can deteriorate cardiac structure and function, which may lead to heart failure in the absence of coronary atherosclerosis and hypertension. However, the multifactorial nature of the disease remains incompletely understood. The present investigation showed that inhibition of MEF2A had a protective role in cardiac function associated with the mechanisms of EndMT and improved hyperglycemic-induced cardiac fibrosis. This is the first report demonstrating that MEF2A inhibition alleviates hyperglycemic-induced EndMT and diabetic myocardial dysfunction.

Myocyte enhancer factor 2 (MEF2) represents the second class of transcriptional factors that regulate expression of many muscle-specific, growth factor-induced, and stress-induced genes [15]. MEF2A is a key nuclear mediator that may participate in the pathological remodeling and accumulation of focal fibrosis in hypertrophic cardiomyopathy [16, 17]. Indeed, a recent study demonstrated that characterization of MEF2A knock-out mice revealed severe myofibrillar defects in cardiac muscle, indicating a requirement for MEF2A in cytoarchitectural integrity [18]. However, another study showed that MEF2A overexpression is sufficient to induce cardiac hypertrophy, and dominant negative inhibition of MEF2A signaling blocked cardiomyocyte hypertrophy [19, 20]. In our present study, MEF2A expression was upregulated in the diabetic myocardium. MEF2A inhibition improved cardiac function and remodeling in diabetic mice. Thus, prolonged activation of MEF2A-dependent genes in myocytes may become maladaptive, contributing to pathological remodeling and accumulation of focal fibrosis in diabetes-induced cardiomyopathy. However, the role of MEF2A in diabetic myocardial fibrosis in endothelial cells is still unknown.

The most important pathological feature of DCM is hyperglycemia-induced excess production of ECM, mainly collagen types I and III, which can alter the structure and function of the heart [21]. The immunohistochemistry and western blot analysis results showed that knocking down MEF2A made a significant reduction in collagen deposition and expression of collagen I and III in the perivascular and intramyocardial regions of the diabetic mouse myocardium. Thus, hyperglycemia leading to MEF2A activation is an essential mechanism that may contribute to myocardial remodeling and fibrosis in DCM, and silencing MEF2A may have therapeutic potential in ameliorating these processes.

Chronic hyperglycemia is an important initiator of EndMT [5]. Recently, the contributions of EndMT to cardiac fibrosis have been reported [2, 22]. EndMT can contribute to the progression of multiple diseases, including diabetic cardiomyopathy, diabetic nephropathy, and hypertensive cardiomyopathy [5, 23, 24]. In the present study, we observed decreased levels of the endothelial marker CD31 and VE-cadherin with HG treatment and increased expression of the fibroblast markers FSP-1 and α-SMA. We then observed colocalization of the endothelial marker CD31 and fibroblast markers FSP-1 and α-SMA in DM arteries. The disaggregated endothelial cells start to alter their structure, exhibit a decrease in endothelial marker expression, and acquire mesenchymal characteristics. This cascade of events reveals the importance of preserving endothelial cell integrity by suppressing MEF2A activity, thereby preventing the initiation of DM-induced EndMT. We further observed that MEF2A plays an important role in these endothelial cells that are undergoing phenotypic transition and beginning to acquire fibroblast characteristics, a process called EndMT in the late stages of DM. Our results indicated that elevated levels of MEF2A may be a trigger of hyperglycemia-induced EndMT and as a profibrotic factor in diabetes-induced cardiomyopathy.

Similar to other published reports [2, 5], we observed that only ~20% to 30% of fibroblasts coexpressed both endothelial markers and fibroblast markers in the hearts of diabetic mice, and we did not evaluate the exact contribution of proliferating resident mesenchymal cells and circulating bone marrow–derived fibroblasts to cardiac fibrosis, which is a limitation in our study. MEF2A is primarily considered to be a transcription factor having a common amino-terminal DNA binding domain and playing pivotal roles in cardiac, muscle, and neuron gene expression [25]. However, previous studies have demonstrated that MEF2A exhibits regionally specific cytoplasmic expression in rodent forebrain [26].

To further evaluate the functions of MEF2A under hyperglycemic conditions *in vitro*, we examined the subcellular localization of MEF2A in HUVECs and clearly showed that HG induced higher expression levels of MEF2A and translocation into the cytoplasm from the nucleus. Moreover, HG only increased the activity of MEF2A (phosphorylation) in the nucleus. In line with this observation, the results of the western blot analysis showed a similar result. HG may enhance MEF2A transcriptional activation and regulate diabetic symptoms. The major MAPK signaling cascades ERK1/2, JNK, and p38 MAPK are strongly activated by hyperglycemia and MEF2Ais a nuclear target for the p38MAPK signaling pathway [27, 28]. In our study, we found that p38 MAPK inhibition decreased MEF2A transcriptional activity in the nucleus of HUVECs with high glucose medium (HG) than normal medium (NG) treatment. Thus, the HG-MAPK-MEF2A pathway is an essential mechanism that may contribute to myocardial remodeling and fibrosis in DCM. MEF2A may mediate cardiac remodeling via multiple mechanisms depending on the underlying pathological condition. The TGF-β superfamily and the Smads with their downstream receptors and signal transducers are the major regulators of EndMT, and they are the main reasons for fibroblast activation, which sequentially leads to myocardial fibrosis and diastolic dysfunction [29–31]. We found that MEF2A silencing could markedly inhibit HG-induced Smad2 expression and activity and also attenuated HG-induced EMT in HUVECs. These results contrast with a previous study demonstrating the function of Smad2 as a co-modulator for MEF2 transcriptional regulatory proteins [13].

In summary, we illustrate that cardiac MEF2A silencing may protect against cardiac fibrosis and improve myocardial function in diabetic mice by regulating EndMT. The mechanism may be involved with the translocation of MEF2A to the cytoplasm and interactions with p38MAPK and Smad2. Given the cardioprotective effects of MEF2A silencing, MEF2A may be a potential therapeutic target for diabetic heart diseases.

## MATERIALS AND METHODS

### Construction of lentiviral-mediated MEF2A interference vector

A lentivirus vector containing a green fluorescent protein (GFP) reporter and a U6 promoter upstream of the cloning site was used for cloning short-hairpin RNAs (shRNAs). The target sequence for *MEF2A*-homo interference was 5′-GCAGCCAGCTCAACGTTAACA-3′ and the negative control sequence was 5′-TTCTCCG AACGTGTCACGT-3′. The target sequence for MEF2A- mus interference was 5′-GCTTGCCACCTCAGAAC TTCT-3′ and the negative control sequence was 5′-TTCT CCGAACGTGTCACGT-3′.

### Animal model and experimental protocol

C57BL/6J wild-type (WT) male 8-week-old mice (23–28 g) were used for the *in vivo* experiments. Type 1 diabetes mellitus was induced by intraperitoneally injections of streptozotocin toxin (STZ; Sigma, St. Louis, MO) dissolved in citrate buffer (pH 4.5) at 60 mg/kg body weight for 5 consecutive days. Control mice (n=25) were injected with citrate buffer only. Mice with randomly measured glucose levels of 20mmol/L 7 days after STZ injection were considered diabetic. Blood glucose was measured using an Accu-Check Active glucometer (Roche). The diabetic mice did not receive any insulin treatment. Thirteen weeks post-STZ injection, the diabetic mice were randomly divided into 3 groups: diabetes mellitus (DM) (n=30), lentivirus-mediated green fluorescent protein of MEF2A interference NC (LV-GFP[−]) (n=30), and lentivirus-mediated MEF2A interference (LV-MEF2A[−]) (n=30). Lentivirus was administered directly to the heart by intramyocardial injection.

The salient steps of delivering lentivirus into the left ventricular wall of the mouse involve administration of anesthesia, intratracheal intubation, incision to open the chest and expose the heart, and delivery of lentivirus by a sterile 30-gauge needle and a precision microliter syringe. For treatment, an amount of 1×107 UT / 30μl of lentivector with MEF2A shRNA or the same volume of lenti-vehicle were injected into 3 sites of the left ventricle. The recombinant lentivirus vector containing a green fluorescent protein (GFP) reporter for measuring transfection efficiency (Supplementary Figure S1A)

All mice were given free access to a normal diet and water. Mice were humanely euthanized and evaluated after twenty-one weeks post-STZ injection. All experiments conformed to the Guide for the Care and Use of Laboratory Animals published by the US National Institutes of Health and Shandong University.

### Cell culture and RNA interference

Human umbilical vein endothelial cells (HUVECs) were purchased from American Type Culture Collection. Cells were grown to confluence in endothelial cell medium (ECM) supplemented with 5% fetal bovine serum and 1% endothelial cell growth supplement. Cells were cultured in a humidified 5% CO_2_ incubator at 37°C and used between the fourth and sixth passages. Cells were treated with 5 or 33 m mol/L d-glucose. The medium was changed every 48 h for 5 consecutive days. Before glucose treatment, the HUVECs were infected with lentivirus at a multiplicity of infection (MOI) of 10 for 24h. For Smad2 inhibition, cells were transfected with small interfering RNA (siRNA) of Smad2 or a nontarget gene using Lipofectamine 2000 reagent (Invitrogen) according to the manufacturer's instructions. Optimal knockdown of Smad2 was obtained by 4h incubation with siRNA.

### Cardiac function measurement

Cardiac diameter and function was measured under 2.0 % isoflurane anesthesia by transthoracic echocardiography using Vevo770 imaging system (Visual Sonics, Toronto, Canada) with a 10-MHz probe. M-mode tracing was recorded at the level of the papillary muscles. Lift ventricular end-diastolic dimension (LVEDD), left ventricular end-systolic dimension (LVESD), and left ventricular diastolic posterior wall thickness (LVPWd) were measured. Percentage of left ventricular ejection fraction (LVEF) was calculated as 100×[(LVvol_d_ – LVvold_s_)/LVvol_d_] and percentage left ventricular fractional shortening (LVFS) was calculated as 100×[(LVEDD – LVESD)/LVEDD]. Pulsed-wave Doppler echocardiography was used to measure the ratio of peak early diastolic ventricular filling velocity to peak atria filling velocity (E/A). And the ratio of diastolic mitral annulus velocities (E'/A') were measured in tissue Doppler imaging. E/E' ratio was calculated.

### Endothelial cells isolated from the heart

Hearts were explanted twenty-one weeks post-STZ injection and cardiac cells were dissociated using a gentle MACS Dissociator (Miltenyi Biotec) as described by the manufacturer. Endothelial cells were isolated using positive selection by magnetic affinity cell sorting using a CD146 antibody (Miltenyi Biotec) and were then used for RNA isolation and RT-PCR.

### Histological analysis

After the physiological analysis, mice were sacrificed for sections. To examine cardiac fibrosis, heart sections were stained with Masson's trichrome (MTC) and Picrosirius red. The intramyocardial and perivascular region fibrotic area was measured from all groups in every 5 randomly chosen views of each sample and analyzed by the Image-Pro Plus 6.0 program. Perivascular fibrosis was calculated as the ratio of the fibrotic area surrounding the vessels to the total vessel area [32].

### Immunofluorescence microscopy

Expression and localization of target genes were observed using immunofluorescence methods. Paraffin-embedded mouse heart sections and cultured HUVECs were incubated with antibodies (mouse anti-MEF2A 1:100, rabbit anti-CD31 1:200, rabbit anti-VE-cadherin 1:400, mouse anti-FSP-1 1:50, mouse anti-α-SMA 1:200, rabbit anti-p38 MAPK 1:200, rabbit anti-Smad2 1:400) overnight at 4ºC. The following day, the tissue sections and cells were incubated with the appropriate secondary antibodies (Alexa Fluor 488 and 549 goat anti-rabbit; Alexa Fluor 549 and 488 anti-mouse 1:100–1:400, CyTM5 goat anti-rabbit, anti-mouse 1:200) for 30 min at 37ºC. All sections were counterstained with 4′-6-diamidino-2-phenylindole (DAPI, Invitrogen). Specific fluorescence was acquired by laser-scanning confocal microscopy (LSM710, Carl Zeiss, Germany). The number of CD31+/ S100A4+ and CD31+/α-SMA+ cells was measured by z-stack image analysis, which confirmed the specific overlay of CD31+/S100A4+ and CD31+/α-SMA+ cells in at least 10 high-power (×630) fields/sections by two independent observers blinded to the origin of the slides [5].

### Co-Immunoprecipitation (CO-IP)

For immunoprecipitation of MEF2A and Smad2, HUVECs were lyzed in Lysis Buffer (P0013) containing PMSF. Supernatant was incubated with MEF2A, Smad2 and IgG antibody for 4°C overnight, and incubated with Protein A/G sepharose beads for 6h. Beads were washed with lysis buffer three times, incubated for 5 min at 95°C with 1X loading buffer and subjected to SDS-PAGE and Western Blot analysis.

### Duolink^®^ in situ-proximity ligation assay

HUVECs were fixed in 4% formaldehyde and incubated with antibodies (anti-MEF2A [mouse origin], Smad2 [rabbit origin]) at 4°C overnight. The cells were subsequently incubated with the PLUS and MINUS PLA probes (Duolink® In Situ PLA® Probe Anti-Mouse PLUS Affinity-purified Donkey anti-Mouse IgG (H+L), Sigma, DUO92001; Duolink® In Situ PLA® Probe Anti-Rabbit PLUS Affinity-purified Donkey anti-Rabbit IgG (H+L), Sigma DUO92002) at 37°C for 1h. Following incubation, the cells were washed and incubated in the Ligation-Ligase solution for 30 min at 37°C. Following an additional wash, the cells were incubated in Amplification-Polymerase solution for 100 min at 37°C. The samples were mounted using Duolink In Situ Mounting Medium with DAPI (Sigma, DUO82040), allowed to adhere for 15 min, and analyzed using a confocal microscope.

### RT-PCR

Primer details are shown in Supplementary Table S1. SYBR Green RT-PCR and quantitative assays involved the use of a sequence detector system (IQ5 Real-Time PCR cycler; Bio-Rad Laboratories, CA, USA). Quantitative values were obtained using the threshold cycle (CT) values. Relative mean fold changes in expression were calculated by the 2^−△△CT^ method.

### Western blot analysis

Protein expression was assayed with cell or tissue lysate of equal protein content, which was separated using 10% SDS-PAGE. Proteins were transferred from the gel to a polyvinylidene difluoride membrane and incubated with antibodies for MEF2A (1:1000, Sigma), CD31 (1:1000, Sigma), VE-Cadherin (1:1000, Cell Signaling), S1004A/FSP-1(1:100, Abcam), vimentin (1:1000, Sigma), α-SMA (1:1000, Abcam), p38MAPK (1:1000, Abcam), phospho-p38MAPK (1:1000, Abcam), Smad2 (1:1000, Abcam), phospho-Smad2 (1:1000, Abcam), or GAPDH 1:5000, Sigma), followed by horseradish peroxides-conjugated goat anti-rabbit (1:5000) or goat anti-mouse IgG (1:5000) (Santa Cruz). Proteins were visualized using an enhanced chemiluminescent reagent (Amersham Pharmacia Biotech) plus WB detection system (Amersham-GE Healthcare). Blots were quantified by densitometry using Image J (NIH Image).

### Statistical analysis

Results are presented as mean ± SEM. Statistical analyses were performed using the Student's *t*-test for direct 2-group comparisons and the Tukey-Kramer test following significant one-way ANOVA test for multiple-group comparisons. Data analysis was carried out by SPSS v16.0 software (SPSS Inc., Chicago, IL) on the results of at least 3 independent experiments. A value of *p* <0.05 was considered statistically significant.

## SUPPLEMENTARY FIGURES AND TABLE


