# Supplementary material for: Inhibition of myocyte-specific enhancer factor 2A improved diabetic cardiac fibrosis partially by regulating endothelial-to-mesenchymal transition
Source: Oncotarget. 2016 Apr 20;7(21):31053–66. doi: 10.18632/oncotarget.8842 (PMC5058738; doi:10.18632/oncotarget.8842)
Supplement: Supplementary file 1 [file oncotarget-07-31053-s001.pdf]

SUPPLEMENTARY FIGURES AND TABLE

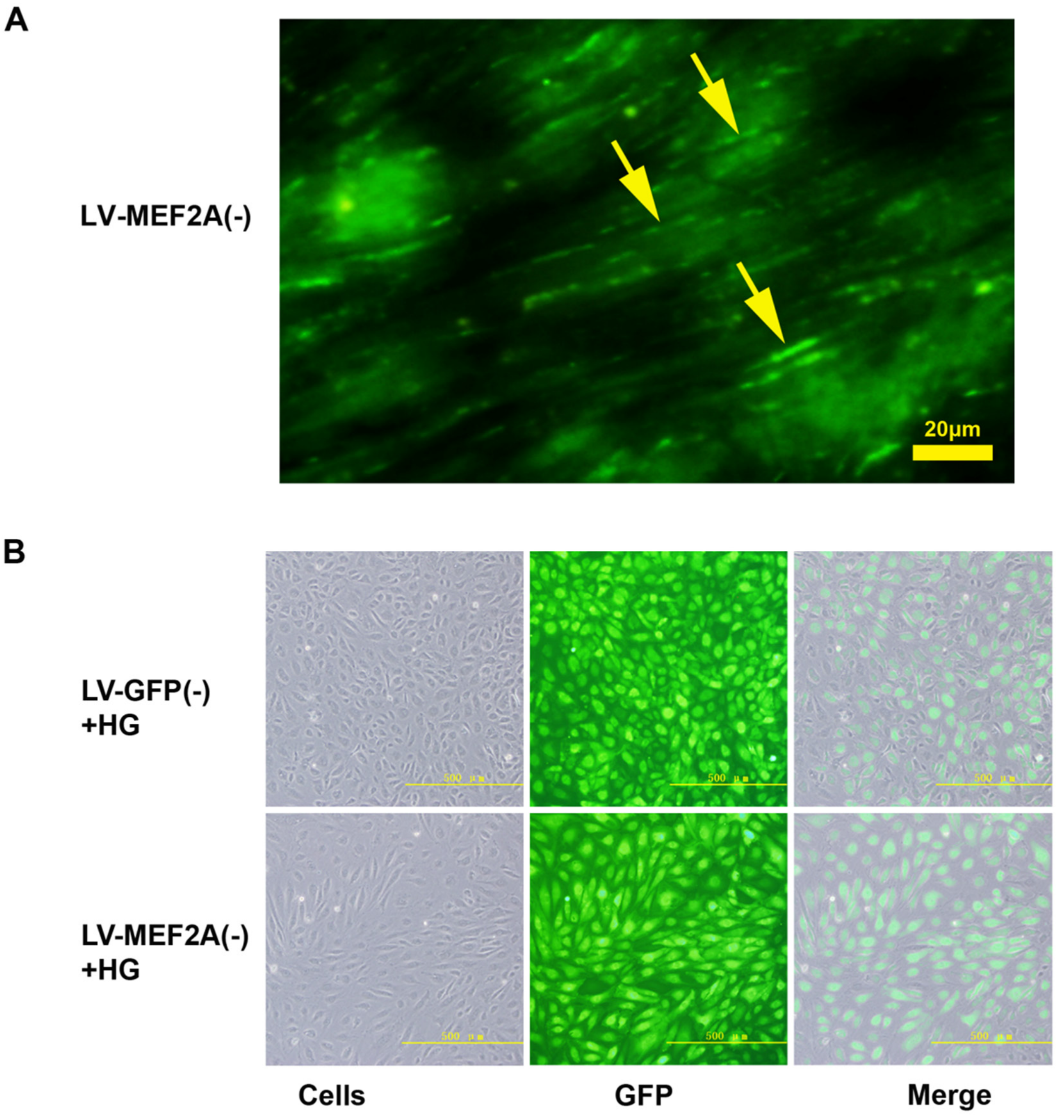

Supplementary Figure S1: Fluorescence microscopy of GFP-labeled transfection efficiency in mouse myocardial tissue and HUVECs. A. GFP immunofluorescence images of mouse myocardial tissue in MEF2A knockdown group. The arrowhead points indicate locations at which GFP is expressed (scale bars=20μm); **B.** Representative photograph of GFP-labeled scramble transfection efficiency of MEF2A knockdown in HUVECs (scale bars =500μm).

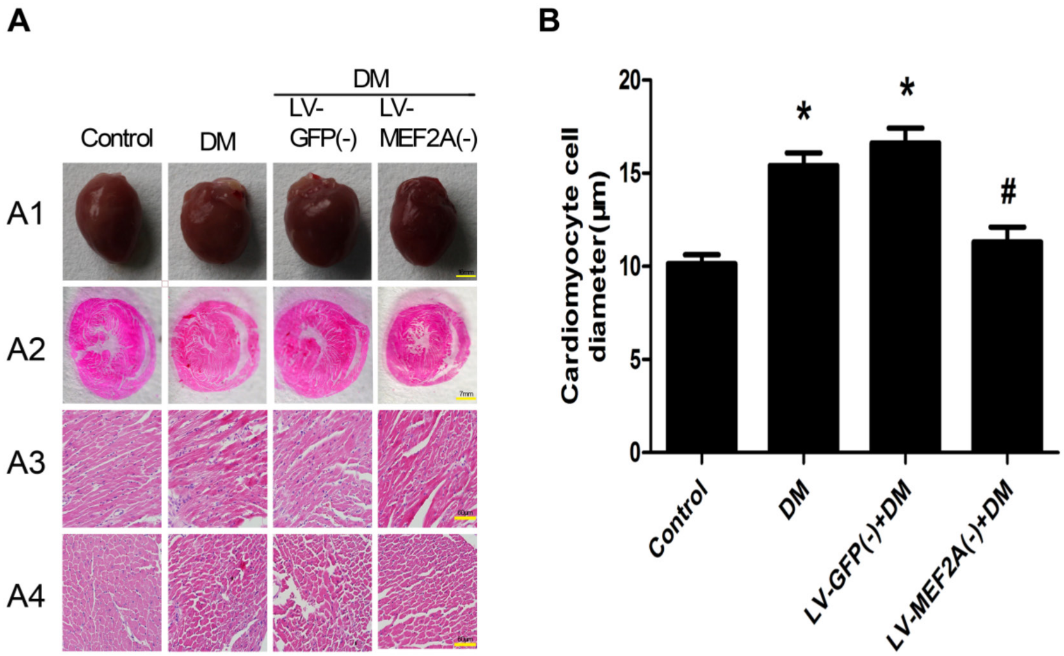

Supplementary Figure S2: Myocardial pathology features and cardiomyocytes cell diameter in control group and STZ-induced diabetes group. A. Myocardial pathology features. (A1) Heart size (scale bars =60mm); (A2) Cardiac histological cross-sectional anatomy at the papillary muscle level (scale bars = 7mm); (A3) Cardiac hematoxylin and eosin staining (H&E) of left ventricular (LV) longitudinal sections (scale bar=60 µm); (A4) Cardiac H&E staining of LV transverse sections (scale bar = 60 µm); **B.** Quantitative analysis of cardiomyocyte cell diameter. Data are expressed as the mean±SEM; n=6 to 8 mice for each group; *$p< 0.05$ vs. control group, #$p< 0.05$ vs. vehicle treatment group.

Supplementary Table S1: PCR primer sequences

Gene	Sense	Antisense
Human MEF2A	5′-AACCCAGGGAGTTCACTGGT-3′	5′-TGTGAGGTCTGTAGTGCTCAA-3’
Mouse MEF2A	5′-GGAGCTGGAAATAGTCCTGTGG-3′	5′-GGGAGCTTTGTAGGCATGACTT-3
Human VE-Cadherin	5′-AAGCGTGAGTCGCAAGAATG-3′	5′-TCTCCAGGTTTTCGCCAGTG-3′
Human CD31	5′-AACAGTGTTGACATGAAGAGCC-3′	5′-AACAGTGTTGACATGAAGAGCC-3′
Human FSP-1	5′-GATGAGCAACTTGGACAGCAA-3′	5′-CTGGGCTGCTTATCTGGGAAG-3′
Human α-SMA	5′-CACAATGTGCGACGAAGACG-3′	5′-CTCTCTTGCTCTGAGCCTCG-3′
Human β-actin	5′-GAGCTACGAGCTGCCTGACG-3′	5′-CCTAGAAGCATTTGCGGTGG-3′
Mouse β-actin	5′-CACTGTGCCCATCTACGA-3′	5′-GTAGTCTGTCAGGTCCCG -3′
